# Comparing full-body flywheel eccentric protocols: cluster set vs. traditional training on muscle damage, arterial stiffness, jump performance, grip strength, cognitive, and perceptual responses

**DOI:** 10.5114/biolsport.2026.158673

**Published:** 2026-04-13

**Authors:** Ming-Chia Weng, Chih-Yuan Fang, Hsi-Hsun Su, Eisuke Ochi, Maximillian J. Nelson, Hunter Bennett, Thomas P. Wycherley, Xiang Dai, Che-Hsiu Chen

**Affiliations:** 1 Department of Physical Education, Chinese Culture University, Taipei, Taiwan; 2 Department of Sport Performance, National Taiwan University of Sport, Taichung, Taiwan; 3 Department of Rehabilitation, Feng Yuan Hospital of the Ministry of Health and Welfare, Taichung, Taiwan; 4 Graduate School of Sports & Health Studies, Hosei University, Tokyo, Japan; 5 Alliance for Research in Exercise, Nutrition, and Activity (ARENA), University of South Australia, Australia

**Keywords:** Recovery, Fatigue, Prevention, Injury, Damage, Preservation, Lengthening

## Abstract

The study investigated the acute effects of traditional (TRAD) and cluster (CLUS) set full-body flywheel eccentric exercise (FW) protocols on muscular performance, damage, vascular responses, and cognitive function. Twenty-four well trained male team sport athletes participated in this randomized controlled trial. Participants were randomly assigned to TRAD or CLUS FW protocols. FW protocols consisted of four sets of eight repetitions for four exercises (squat, high pull, bent over row, and calf raise), with 120 seconds of rest between sets. Measured variables included creatine kinase (CK), muscle soreness, muscle and tendon stiffness, countermovement jump (CMJ), handgrip strength, blood pressure, cardio-ankle vascular index (CAVI), anklebrachial index (ABI), heart rate, cognitive function, and fatigue. These assessments were conducted pre-exercise, immediately post-exercise, and at 24 h and 48 h post-exercise. Both protocols showed similar power outputs and cognitive improvement over time (p < .05). Handgrip strength declined in both groups, while perceived fatigue and muscle soreness increased (p < .05). However, the CLUS exhibited lower CK levels, lower quadriceps stiffness at 24 and 48 h post-exercise, and lower patellar tendon stiffness immediately post-exercise, compared to the TRAD (p < .05). Greater vascular fluctuations, particularly in diastolic blood pressure (DBP) and CAVI, were observed in the TRAD group (p < .05). These findings suggest that a cluster set protocol for full-body flywheel eccentric exercise may be advantageous for recovery compared to a traditional set protocol in well trained male team sport athletes.

## INTRODUCTION

Eccentric overload training utilizing flywheel devices (FW) has garnered considerable attention in recent years for its capacity to induce substantial improvements in muscle strength, hypertrophy, and neuromuscular efficiency [1]. Unlike traditional weight-based resistance training, FW systems provide variable resistance and allow users to generate high eccentric loads, which stimulate unique muscular adaptations. However, the high mechanical stress imposed during eccentric phases is commonly associated with pronounced delayed onset muscle soreness (DOMS), transient declines in performance, increased muscle stiffness, and elevated markers of muscle damage [2, 3].

While the efficacy of FW eccentric training in enhancing muscular development is well documented, optimizing its efficiency and tolerability remains a challenge particularly in contexts where managing fatigue, minimizing injury risk, and supporting recovery are essential. Despite their well-known anabolic and neuromuscular benefits, traditional FW eccentric exercises can elicit considerable acute muscle damage, limiting their applicability within high-frequency training schedules or in-season programs. For example, Raeder et al. reported elevated blood lactate levels and ratings of perceived exertion following four sets of eight repetitions of the flywheel squat [4]. To mitigate the adverse effects commonly seen in eccentric training, cluster set training, which introduces brief intra-set rest periods (typically 10–30 seconds), has been proposed as an effective strategy to reduce acute fatigue and mechanical strain. In contrast to traditional continuous sets, cluster set configurations have been shown to help maintain power output across a given set, facilitate the maintenance of consistent exercise technique, and potentially reduce the physiological stress associated with high-intensity resistance training [5]. This training approach preserves movement velocity, lowers perceived exertion, and minimizes metabolic accumulation, thereby enhancing overall training quality [5]. Moreover, recent findings suggest that cluster sets may play a role in modulating post-exercise inflammation and oxidative stress, potentially providing protective effects on muscle tissue and the central nervous system [6]. Supporting this, evidence also indicates that cluster sets can reduce delayed onset muscle soreness (DOMS) and subjective fatigue when compared to traditional set structures [7]. Despite the growing adoption of cluster sets in barbell-based strength programs, their application within eccentric-dominant FW training remains largely unexplored and warrants further investigation [8].

In parallel, there is increasing recognition of the multisystemic benefits of resistance training, particularly its influence on cognitive function, vascular health, and autonomic regulation factors that are critically relevant in both aging populations and high-performance athletes exposed to cumulative physiological stress. Resistance training has been associated with improvements in executive function and attentional control, potentially mediated by enhanced cerebral perfusion and neurochemical modulation [9–11]. In particular, eccentric resistance exercise appears to elicit superior acute cognitive benefits, likely due to greater cortical engagement and the unique motor control demands required during eccentric contractions [12]. Additionally, growing evidence supports its favorable impact on cardiovascular health, including reductions in blood pressure, arterial stiffness, and improvements in autonomic control [13, 14]. For instance, Wang et al. investigated a whole-body, high-intensity, shortrest resistance training protocol involving back squats, bench press, and deadlifts at 75% of one-repetition maximum (1RM), and reported significant postexercise hypotension (PEH), including reductions in blood pressure, mean arterial pressure, and ankle–brachial index [13]. Moreover, both acute and long-term iso-inertial FW eccentric resistance exercise did not impair cardiovascular responses or brachial artery vasodilation capacity [14, 15]. In a 10-week comparison of whole-body traditional isotonic resistance training and isoinertial FW eccentric training (including exercises such as back squat, bench press, deadlift, row, and bicep curl), the study found that the isotonic protocol led to impaired cardiovagal function and blood pressure reactivity, whereas the FW eccentric training preserved cardiovascular autonomic regulation in healthy, active young adults [14].

Notably, there is currently a lack of research investigating the effects of full-body eccentric overload protocols using FW devices. This gap limits our understanding of how systemic neuromuscular and physiological responses may differ when multiple major muscle groups are eccentrically trained within the same session. The present study is among the first to implement a full-body FW eccentric training protocol, incorporating both upper and lower limb movements, thereby broadening the application scope and relevance of eccentric overload training in both athletic and clinical domains. Therefore, the present study aims to comprehensively compare the acute effects of whole-body traditional versus cluster set FW eccentric training on a broad range of physiological and performance indicators, including muscle damage markers, muscle soreness, muscle stiffness, countermovement jump (CMJ), hand grip strength, cognitive function, blood pressure, and arterial stiffness markers. This study seeks to contribute novel insights into how manipulating the structure and scope of FW eccentric training including full-body implementation may enhance performance outcomes while promoting neurovascular health and reducing the negative consequences typically associated with eccentric overload. Findings may support the development of evidence-based, sustainable training paradigms applicable to athletes, clinical populations, and health-conscious individuals. We hypothesized that CLUS would result in fewer negative effects on muscle damage, arterial stiffness, muscle strength, as well as cognitive and perceptual responses compared to TRAD.

## MATERIALS AND METHODS

### Experimental approach to the problem

This study employed a randomized controlled trial with a betweengroup design to compare the effects of cluster set versus traditional full-body FW eccentric training protocols on markers of muscle damage, strength, cognitive function, cardiorespiratory parameters, and perceptual responses. Participants attended the laboratory over two consecutive weeks. In the first week, they underwent familiarization sessions for both the testing procedures and the full-body FW eccentric training protocols. Participants (n = 24) were randomly assigned to either a cluster set group (CLUS) or a traditional group (TRAD) (n = 12 per group), with stratified randomization based on baseline average power output during a flywheel squat exercise. Allocation concealment was maintained using sequentially numbered, opaque, sealed envelopes, which were prepared by an independent researcher and opened only after baseline assessments were completed.

In the second week, all participants performed a single bout of full-body FW eccentric training following a standardized 10-minute warm-up, consisting of 5 minutes of jogging and 5 minutes of dynamic stretching. All dependent variables were assessed at four time points: baseline (pre-test), immediately after training (post-test), 24 hours post-training, and 48 hours post-training. To minimize the influence of diurnal variation, all measurements were conducted at the same time of day for each participant. The outcome variables and the order of assessment were as follows: plasma creatine kinase (CK) activity, heart rate (HR), perceptual responses, cognitive function, perceived muscle soreness, muscle and tendon stiffness, countermovement jump (CMJ) height, handgrip strength, systolic blood pressure (SBP), diastolic blood pressure (DBP), cardio-ankle vascular index (CAVI), ankle-brachial index (ABI), and mean arterial pressure (MAP).

### Participants

The present study involved 24 well trained male team sport athletes (mean ± SD, age = 24.38 ± 3.66 years; height = 175.27 ± 5.79 cm; and body mass = 67.13 ± 9.55 kg). The research involved athletes from 3 different sport disciplines: baseball (n = 6), rugby (n = 9), and handball (n = 9) players. People who had ankle, knee, hip, lower-back, or hamstring muscle-related injuries in the 12 months prior to the study were not eligible for inclusion. All participants were national Division 1 athletes who trained five times per week with 3–4 h (including rest periods) spent in each training session. Each participant provided informed consent before commencing in the study. Participants refrained from vigorous physical activities and training at least 5 days before experimental visits. Additionally, all participants were in their off-season during the study period. On all the experimental visit days, they were not allowed to consume any food or supplements that contained alcohol and/or caffeine or take anti-inflammatory medicine. All experimental procedures in this study were performed in accordance with the Declaration of Helsinki and approved by the Institutional Review Board (No.: IRB-202300007A3).

### Full-body flywheel eccentric training protocols

**FIG. 1:**
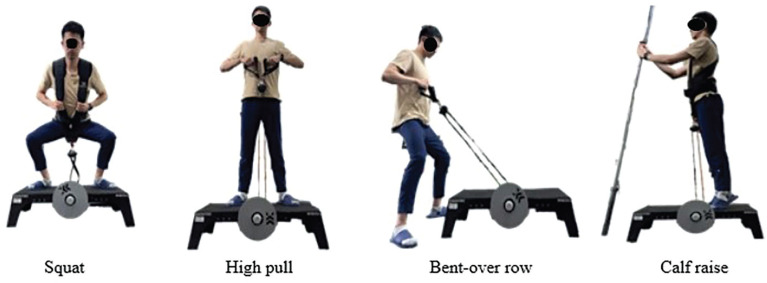
Full-body flywheel eccentric exercise (FW) protocols.

The full-body FW eccentric training protocols utilized a flywheel device (kBox4, Exxentric AB™, Bromma, Sweden), with the resistance set at 0.075 kg · m^2^. Training exercises included squat, high pull, bent over row, and calf raise (Figure 1). Participants were divided into two groups. The TRAD group performed four sets of eight repetitions for each exercise, with 120 seconds of rest between sets. The first two repetitions in each set were executed submaximally to generate momentum, followed by six maximal-effort repetitions.

Similarly, the CLUS group also completed four sets per exercise; however, each set was divided into two clusters of four repetitions, separated by a 20-second intra-set rest, with 120 seconds of rest between sets. In each cluster, the initial two repetitions were performed submaximally to acquire momentum, while the subsequent two repetitions were performed maximally, resulting in a total of 4 submaximal and 4 maximal repetitions per set. We instructed participants to apply maximum force during the concentric phase and resist the braking during the eccentric phase. All exercises were monitored in real time using the proprietary KMeter software, which recorded strength training metrics such as average power (defined as all repetitions in the set both concentric and eccentric phase), concentric peak power, and eccentric peak power outputs.

### Outcome Measures

#### Serum creatine kinase activity

Approximately 5 ml of venous blood was drawn from the cubital fossa region of the arm by using a standard venipuncture technique and was centrifuged for 10 minutes to extract plasma. The plasma samples were stored at -80 °C until analysis. CK activity was measured using an automated clinical chemistry analyzer (Model Elecsys 2010, F. Hoffmann-La Roche, Tokyo, Japan) and commercial test kits (Roche Diagnostics, Indianapolis, IN).

#### Muscle soreness

Muscle soreness was assessed using a 100-mm visual analog scale, with 0 mm being “no soreness” and 100 mm being “extremely sore”. The participants drew a point on the line indicating their muscle soreness when the tester palpated over the biceps brachii, latissimus, biceps femoris, quadriceps, and gastrocnemius muscles in a relaxed standing position. All measurements were performed by the same tester, and semipermanent ink marks ensured consistent palpation sites across sessions.

#### Muscle and tendon stiffness

Soft tissue mechanical properties were assessed using the Myoton PRO device (Myoton AS, Tallinn, Estonia). This non-invasive instrument delivers a brief mechanical impulse (0.4 N for 15 ms) to the surface of the skin and records the resulting oscillations of the underlying soft tissue. From these oscillations, muscle stiffness (N/m) is quantified [16]. Measurements were taken in a relaxed supine position for the quadriceps, biceps brachii, and patellar tendon, and in a prone position for the latissimus dorsi, biceps femoris, gastrocnemius, and Achilles tendon. Stiffness was assessed at the midpoint (1/2 of muscle or tendon length) under resting conditions. Each muscle or tendon was measured three times, and the average of the three readings was used for analysis. This test has an ICC of 0.85–0.91, suggesting high test-retest reliability [16].

#### Countermovement jump

The participants stood on a jump mat (Smart Jump, Fusion Sport, Brisbane, Australia) with their hands on their hips and quickly bent down before jumping maximally, keeping their legs straight throughout. The flight time was used to estimate the jump height. All participants performed three times, with a 60-second rest between trials, with the height of each being recorded (centimeters). The highest height recorded was used for analysis. This test has an ICC greater than 0.9 [17].

#### Handgrip strength

Handgrip strength was measured using a handheld dynamometer (TTM, Smedley’s dynamometer), with the grip span adjusted according to the participants’ hand size. Participants were instructed to hold the dynamometer with their arm relaxed and extended downward. The dominant hand contraction was held for 5 seconds and repeated three times, with a 60-second rest between trials. The highest grip strength value was recorded for analysis. Verbal encouragement was also used to motivate the participants during the tests.

#### Hemodynamic and vascular stiffness

A vascular screening system (VaSera VS-2000, Fukuda Denshi, Tokyo, Japan) was used to measure SBP, DBP, CAVI, and ABI. The participants were instructed to quietly rest in the supine position for at least 15 min. Occlusion and cuffs were wrapped around the bilateral brachial and tibial arteries. The HR was collected using a heart rate monitor (Polar V800, Electro Oi, Finland). MAP was calculated using the following formula: (SBP + [2DBP])/3 [18].

#### Cognitive function, and perceptual responses

Before and after the FW exercise session, participants completed a questionnaire to evaluate their subjective feelings of focus, energy, alertness, and fatigue using a 5-point Likert scale, in accordance with previously established methods [19]. Participants were asked to verbally rate their perceived states on a scale from 1 to 5, where 1 = very low, 2 = low, 3 = average, 4 = high, and 5 = very high.

To assess cognitive function, a computerized version of the Stroop color and word task was administered. Two types of trials were used: congruent (word meaning and font color matched) and incongruent (word meaning and font color mismatched). Participants were asked to quickly respond with the color of the text, not the name of the color written, by pressing the corresponding-colored key on their keyboard. Each condition consisted of 5 trials, and reaction times (in milliseconds) were recorded and analyzed [10, 20].

### Statistical analyses

A priori power analyses using G*Power 3.1 indicated that a minimum sample size of 20 participants was required for intergroup comparisons to achieve a statistical power of 0.80 for detecting differences in muscle damage markers and muscle performance variables [2, 3]. All statistical tests were conducted using IBM SPSS Statistics 24.0 (IBM Corp., Armonk, NY). The Shapiro-Wilk test was used to confirm normality of the data. In addition, t-tests were conducted to compare the power output values obtained during FW eccentric exercises between the TRAD and CLUS groups. If the data were normally distributed, analysis of variance (ANOVA) was conducted. To evaluate the outcome variables, a 2 × 4 mixed-design ANOVA was used, with two experimental protocols (TRAD vs. CLUS) and four time points (pre-test, post-0, post-24, and post-48 hours after eccentric exercise). When a significant interaction effect was detected, post hoc comparisons with Bonferroni correction were applied. Effect sizes were estimated using partial eta squared (η^2^) and interpreted as < 0.06 = small, 0.06–0.13 = medium, and > 0.13 = large [21], and the significance level was set at α = 0.05.

## RESULTS

### FW eccentric exercises

All participants completed the full protocol for their allocated exposure (TRAD or CLUS, including squat, rowing, calf raise, and high pull exercises). No significant differences were found in average, concentric, or eccentric power outputs between TRAD and CLUS protocols across all exercises (p > 0.05) (Table 1).

**TABLE 1:** Mean ± SD power exerted during FW exercises.

	Squat	Bent-over row	Calf raise	High pull
**TRAD**				
Average power (watts)	208.17 ± 112.21	87.27 ± 36.55	83.77 ± 70.43	76.16 ± 26.50
Concentric power (watts)	449.02 ± 275.50	163.48 ± 67.87	182.90 ± 151.68	172.19 ± 66.17
Eccentric power (watts)	495.43 ± 253.37	215.29 ± 100.83	212.58 ± 176.77	274.97 ± 116.07

**CLUS**				
Average power (watts)	169.77 ± 86.11	81.50 ± 44.69	88.23 ± 57.21	90.12 ± 41.69
Concentric power (watts)	416.35 ± 235.52	164.21 ± 99.93	212.33 ± 141.43	213.93 ± 95.97
Eccentric power (watts)	477.17 ± 244.87	209.06 ± 125.28	237.77 ± 179.34	298.15 ± 128.86

TRAD: traditional; CLUS: cluster

### Plasma creatine kinase (CK)

**FIG. 2:**
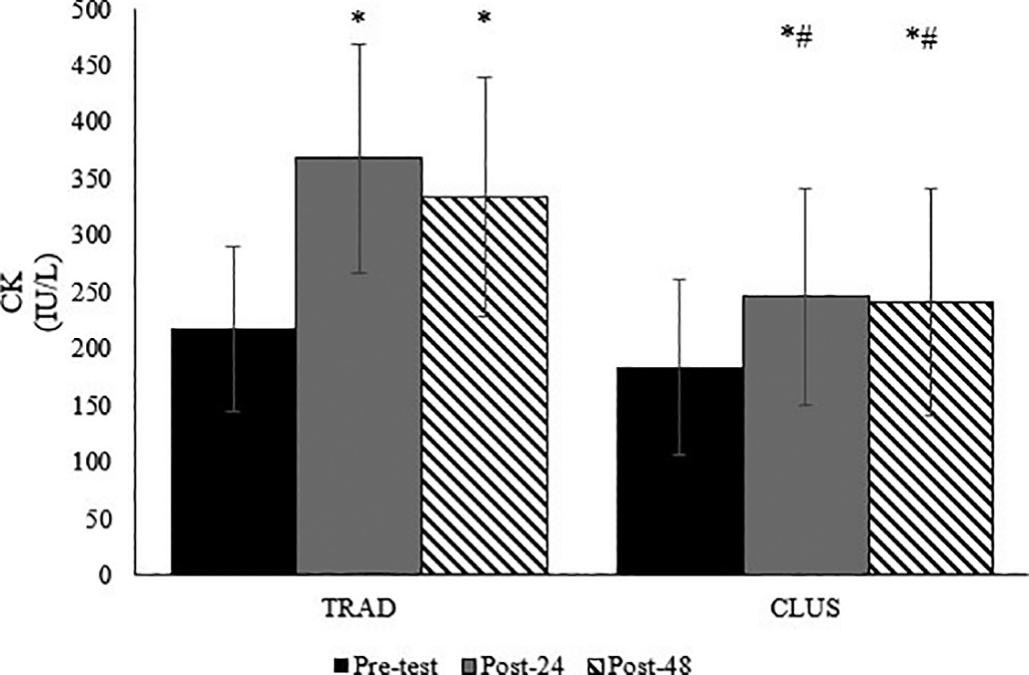
Mean ± SD plasma CK before (pre-test), 1 day (post-24), and 2 days (post-48), after the full-body FW eccentric training protocols. *: Significant difference between pre-test (p < 0.05). #: Statistically significant difference between TRAD (p < 0.05).

There was a time × protocol interaction for plasma CK (F = 5.18; p = .02; partial η^2^ = .19, *large effect*) (Table 2 and Figure 2). Follow-up analyses indicated CK increased from pre-test to 24 hours and 48 hours post-exercise in both TRAD and CLUS groups (p < 0.05). In addition, at post-24 and 48 hours, CK values for CLUS were lower than TRAD protocol (p = .01; p = .04, respectively).

**TABLE 2:** Mean ± SD before (Pre-test), 1 day (Post-24), 2 days (Post-48) after the FW eccentric exercises for plasma CK, and muscle soreness.

		Pre-test	Post-24	Post-48
CK (IU/L)	TRAD	217.58 ± 73.21	367.89 ± 100.57^*^	334.64 ± 105.54^*^
	CLUS	183.25 ± 77.36	245.92 ± 95.51^*#^	240.42 ± 99.85^*#^

Biceps brachii soreness (mm) ^&^	TRAD	5.00 ± 10.00	28.33 ± 24.80	16.67 ± 20.15
	CLUS	5.83 ± 9.96	23.33 ± 17.23	17.50 ± 13.56

Quadriceps soreness (mm) ^&^	TRAD	7.50 ± 9.65	36.67 ± 30.55	25.83 ± 22.75
	CLUS	10.00 ± 14.77	33.33 ± 19.23	26.67 ± 19.23

Latissimus soreness (mm) ^&^	TRAD	9.16 ± 16.21	31.67 ± 22.90	21.67 ± 20.81
	CLUS	13.33 ± 16.69	32.50 ± 20.06	22.08 ± 20.30

Biceps femoris soreness (mm) ^&^	TRAD	10.00 ± 20.44	27.50 ± 24.17	15.00 ± 13.83
	CLUS	10.00 ± 14.77	20.00 ± 13.48	17.50 ± 14.84

Gastrocnemius soreness (mm) ^&^	TRAD	10.00 ± 18.58	24.17 ± 22.09	14.17 ± 13.30
	CLUS	7.50 ± 11.38	24.58 ± 16.71	23.33 ± 21.46

* : Significant difference between Pre-test (*p* < 0.05).

# : Statistically significant difference between TRAD (*p* < 0.05).

& : Statistically significant main effect for time (*p* < 0.05).

### Muscle soreness

No significant interaction between time and protocol was observed for muscle soreness for any of the muscle groups when comparing the TRAD and CLUS protocols (Table 2). A significant main effect of time was found for all muscles, with soreness in the biceps brachii, latissimus dorsi, quadriceps, and gastrocnemius significantly increased from pretest to 24 and 48 hours post-exercise (p < .001). In contrast, biceps femoris soreness significantly increased only from pretest to 24 hours (p < .001), with no further increase at 48 hours.

### Muscle and tendon stiffness

No significant time × protocol interaction was observed for the biceps brachii, latissimus dorsi, biceps femoris, gastrocnemius, or Achilles tendon when comparing the TRAD and CLUS protocols (Table 3).

**TABLE 3:** Mean ± SD before (Pre-test), Post-0, 1 day (Post-24), 2 days (Post-48) after the flywheel eccentric exercises for muscle and tendon stiffness.

		Pre-test	Post-0	Post-24	Post-48
Biceps brachii (N/m)	TRAD	237.66 ± 33.85	241.92 ± 45.42	228.42 ± 31.15	231.92 ± 27.46
	CLUS	236.75 ± 12.64	239.75 ± 29.81	247.33 ± 24.74	263.17 ± 57.86

Quadriceps (N/m)	TRAD	288.33 ± 16.08	307.83 ± 78.37	329.50 ± 23.88^*^	309.50 ± 18.68^*^
	CLUS	278.50 ± 44.45	276.83 ± 34.83	279.00 ± 17.29^#^	278.75 ± 19.67^#^

Latissimus (N/m) ^&^	TRAD	266.25 ± 56.22	286.50 ± 69.68	313.17 ± 68.46	294.92 ± 64.44
	CLUS	302.08 ± 50.85	325.92 ± 55.59	328.92 ± 36.05	336.67 ± 61.81

Biceps femoris (N/m)	TRAD	294.67 ± 50.20	300.67 ± 47.76	292.33 ± 40.65	282.50 ± 53.60
	CLUS	269.75 ± 56.82	278.83 ± 55.64	271.42 ± 47.84	275.33 ± 50.00

Gastrocnemius (N/m)	TRAD	286.50 ± 25.88	293.83 ± 31.43	292.00 ± 28.11	290.50 ± 28.91
	CLUS	297.67 ± 37.73	300. 58 ± 46.13	308.25 ± 50.38	318.92 ± 60.23

Patellar tendon (N/m)	TRAD	740. 58 ± 108.47	793.17 ± 109.18^*^	725.67 ± 106.84^+^	697.08 ± 122.21^*^
	CLUS	677.67 ± 71.76	710.16 ± 77.68^*#^	718.92 ± 97.73^*^	725.42 ± 70.13^*^

Achilles tendon (N/m) ^&^	TRAD	741.83 ± 68.74	808.33 ± 114.11	782.58 ± 148.27	757.00 ± 116.63
	CLUS	725.58 ± 126.87	769.75 ± 95.26	750.50 ± 56.64	769.33 ± 77.07

* : Significant difference between Pre-test (*p* < 0.05).

+ : Significant difference between Post-0 (*p* < 0.05).

# : Statistically significant difference between TRAD (*p* < 0.05).

& : Statistically significant main effect for time (*p* < 0.05).

In contrast, a significant time × protocol interaction was found for quadriceps muscle stiffness (F = 3.01; p = .04; partial η^2^ = .12, *medium effect*), along with a significant main effect of time (F = 3.19; p = .03). Post hoc analysis revealed that in the TRAD group, quadriceps stiffness increased from pre-test to 24 and 48 hours post-exercise (p < .001), while no changes were observed in the CLUS group (p > .05). Additionally, quadriceps stiffness was lower in the CLUS group compared to the TRAD group at both 24 hours (p < .001) and 48 hours (p = .001) post-exercise.

For patellar tendon stiffness, a significant time × protocol interaction was also observed (F = 9.14; p = < .001; partial η^2^ = .30, *large effect*), with a significant main effect of time (F = 5.24; p = .003). Follow-up analysis showed an increase in tendon stiffness from pre-test to immediately post-exercise (Post-0), and a decrease from pre-test to post 48 hours (p = .003) in the TRAD group. In the CLUS group, patellar tendon stiffness increased from pre-test to Post-0 (p = .04), 24 hours (p = .04), and 48 hours (p = .003). Notably, at Post-0, patellar tendon stiffness in the CLUS group was lower than in the TRAD group (p = .04).

### Countermovement jump and hand grip strength

The CMJ and handgrip strength results are presented in the Table 4. No significant interaction between time and protocol was observed for either CMJ or handgrip strength. However, a significant main effect of time was identified for handgrip strength (p = .03), with hand grip strength showing a decrease from pretest to immediately post-exercise (Post-0) (p = .004), suggesting that the exercise protocol induced acute muscular fatigue.

**TABLE 4:** Mean ± SD before (Pre-test), Post-0, 1 day (Post-24), 2 days (Post-48) after the FW eccentric exercises for CMJ, hand grip strength, SBP, DBP, MAP, CAVI, ABI index, and heart rate.

		Pre-test	Post-0	Post-24	Post-48
CMJ (cm)	TRAD	37.95 ± 6.72	37.78 ± 6.30	37.31 ± 5.23	37.42 ± 5.71
	CLUS	38.75 ± 8.86	38.57 ± 8.51	39.05 ± 8.99	40.73 ± 7.96

Hand grip strength (kg)^&^	TRAD	84.70 ± 11.56	82.42 ± 12.54	84.13 ± 11.11	84.00 ± 10.60
	CLUS	88.96 ± 13.63	83.96 ± 12.64	85.54 ± 11.35	86.63 ± 13.05

SBP (mmHg)	TRAD	123.58 ± 10.06	124.67 ± 10.73	123.00 ± 10.90	125.25 ± 10.16
	CLUS	122.58 ± 8.68	123.58 ± 9.20	122.58 ± 9.08	121.50 ± 8.47

DBP (mmHg)	TRAD	70.50 ± 6.79	59.58 ± 5.40^*^	68.92 ± 7.12^+^	70.33 ± 5.42^+^
	CLUS	66.92 ± 5.68	64.72 ± 6.06^#^	67.50 ± 3.42	68.42 ± 3.12

MAP (mmHg)^&^	TRAD	89.20 ± 6.81	82.66 ± 7.05	86.94 ± 7.76	88.64 ± 6.23
	CLUS	85.47 ± 5.33	84.36 ± 5.57	86.97 ± 4.70	88.56 ± 6.69

CAVI	TRAD	5.49 ± 1.45	4.46 ± 1.20^*^	6.08 ± 1.40^+^	5.58 ± 1.07
	CLUS	6.19 ± 1.40	5.98 ± 1.40^#^	5.35 ± 1.25	6.04 ± 1.19

ABI index	TRAD	1.05 ± 0.07	1.06 ± 0.08	1.03 ± 0.14	1.05 ± 0.06
	CLUS	1.10 ± 0.09	1.09 ± 0.08	1.07 ± 0.10	1.08 ± 0.07

Heart rate (bpm) ^&^	TRAD	59.58 ± 9.43	85.42 ± 13.91	60.91 ± 8.50	61.33 ± 9.17
	CLUS	57.25 ± 10.28	84.41 ± 18.83	56.75 ± 10.11	62.58 ± 12.15

* : Significant difference between Pre-test (*p* < 0.05).

+ : Significant difference between Post-0 (*p* < 0.05).

# : Statistically significant difference between TRAD (*p* < 0.05).

& : Statistically significant main effect for time (*p* < 0.05).

### Hemodynamic and vascular stiffness

The results for SBP, DBP, MAP, CAVI, ABI index, and heart rate are presented in the Table 4. No significant time × protocol interaction was observed for SBP, MAP, ABI, or heart rate.

For DBP, a significant time × protocol interaction was also observed (F = 5.66, p = .005, partial η^2^ = .21, *large effect*). Post hoc analysis revealed that in the TRAD group, DBP decreased from pre-test to post-0 (p < .001), and although it subsequently increased in comparison to post-0 at both 24 hours (p = .004) and 48 hours (p < .001) post-exercise, these values were not different from the pre-test baseline. In contrast, no changes in DBP were observed in the CLUS group (p > .05). Notably, at post-0, the DBP value in the CLUS group was higher than in the TRAD group (p = .009).

For the CAVI, a significant time × protocol interaction was also found (F = 3.64, p = .02, partial η^2^ = .14, *large effect*). In the TRAD group, CAVI decreased from pre-test to post-0 (p = .04) and then increased from post-0 to 24 hours (p = .02); however, neither the 24-hour nor 48-hour values differed significantly from the pretest value. No changes were observed in the CLUS group throughout the testing period (p > .05). At post-0, the CAVI value in the CLUS group was higher than that in the TRAD group (p = .009).

In addition, significant main effects of time were observed for both MAP and heart rate (p < .001 for both). When data were collapsed across protocols, MAP decreased from pre-test to post-0 (p = .003), and post-0 MAP was lower than values at 24 hours (p = .02) and 48 hours (p < .001). Heart rate increased from pre-test to post-0 (p < .001) and was elevated at post-0 compared to both 24 and 48 hours (p < .001 for both).

### Cognitive performance and perceptual responses

The results for the Stroop test, as well as subjective ratings of fatigue, focus, energy, and alertness, are presented in Table 5. No significant time × protocol interactions were found for any of these variables. However, significant main effects of time were observed for Stroop congruent time (p < .001), Stroop incongruent time (p < .001), and fatigue (p < .001). When data were collapsed across protocols, Stroop congruent time decreased from pre-test to Post-0 (p = .002), 24 hours (p < .001), and 48 hours (p < .001) post-exercise. Similarly, Stroop incongruent time decreased from pre-test to Post-0 (p < .001), 24 hours (p = .001), and 48 hours (p < .001) post-exercise. In contrast, fatigue increased from pre-test to Post-0 (p = .004) post-exercise.

**TABLE 5:** Mean ± SD before (Pre-test), Post-0, 1 day (Post-24), 2 days (Post-48) after the FW eccentric exercises for Stroop task tests, fatigue, focus, energy, alertness.

Pre-test	Post-0	Post-24	Post-48
Stroop congruent time (ms) ^&^	TRAD	0.92 ± 0.20	0.78 ± 0.14	0.75 ± 0.13	0.77 ± 0.17
	CLUS	0.82 ± 0.22	0.76 ± 0.22	0.68 ± 0.11	0.70 ± 0.23

Stroop incongruent time (ms) ^&^	TRAD	1.00 ± 0.22	0.85 ± 0.15	0.86 ± 0.15	0.81 ± 0.14
	CLUS	0.96 ± 0.32	0.88 ± 0.32	0.88 ± 0.38	0.81 ± 0.28

Fatigue ^&^	TRAD	2.66 ± 0.65	3.42 ± 0.99	3.08 ± 1.16	2.83 ± 1.03
	CLUS	2.75 ± 0.87	3.50 ± 0.80	3.00 ± 1.04	2.25 ± 0.75

Focus	TRAD	3.58 ± 0.90	3.75 ± 0.96	3.66 ± 0.89	3.50 ± 0.90
	CLUS	3.66 ± 0.77	3.33 ± 1.07	3.41 ± 0.51	3.50 ± 0.79

Energy	TRAD	3.33 ± 0.98	3.58 ± 1.24	3.75 ± 0.75	3.67 ± 1.07
	CLUS	3.25 ± 0.75	3.17 ± 1.33	3.33 ± 0.89	3.50 ± 1.00

Alertness	TRAD	3.58 ± 1.08	3.58 ± 0.96	3.50 ± 1.00	3.67 ± 0.98
	CLUS	3.33 ± 1.07	3.08 ± 1.08	3.16 ± 0.83	3.17 ± 0.93

& : Statistically significant main effect for time (*p* < 0.05).

## DISCUSSION

This study investigated whether the CLUS full-body FW protocol could attenuate muscle damage, reduce arterial stiffness, and minimize negative effects on jump performance, grip strength, cognitive function, and perceptual responses. To the best of the authors’ knowledge, this is the first study to compare the effects of the CLUS protocol with a traditional (TRAD) approach across these outcome measures, particularly in the context of full-body FW training. The findings were: (1) Both the TRAD and CLUS FW protocols produced similar acute muscular power responses; (2) The CLUS protocol showed greater efficacy in reducing markers of muscle damage;(3) Quadriceps stiffness increased in the TRAD group; (4) The CLUS group exhibited significantly lower patellar tendon stiffness immediately post-exercise compared to the TRAD group; (5) The CLUS training appeared to preserve arterial function without inducing significant hemodynamic fluctuations or inflammatory responses; and (6) Both protocols improved cognitive function following exercise.

### Neuromuscular power output across protocols

This study implemented four exercises including squats, high pulls, bent over rows, and calf raises each performed for four sets of eight repetitions, with 120 seconds of rest between sets. Although both the TRAD and CLUS groups completed the same training volume, the CLUS group implemented a cluster set configuration in which each set was divided into two mini-sets of four repetitions, &: Statistically significant main effect for time (*p* < 0.05). separated by a 20-second intra-set rest interval. The results revealed no differences in average, concentric, or eccentric power output between the TRAD and CLUS protocols, indicating comparable neuromuscular performance across both conditions. This aligns with existing literature which highlights the potential benefits of clustering strategies in maintaining power under fatigue-inducing conditions. For instance, Nunez et al. showed that rugby players performing high pulls using inertial FW resistance maintained stable power output across six sets of six repetitions when a 20-second rest was provided between sets [22]. Similarly, Sabido et al. reported that a 2-minute inter-set rest preserved power output during FW squats (four sets of 11 repetitions) with light inertia (0.025 kg · m^2^), though this effect diminished under heavier (0.075 kg · m^2^) loads [23]. Moreover, O’Brien et al. [23] reported that in deadlift exercises using FW devices across four sets of 14 repetitions, peak concentric and eccentric power declined after repetition 11 and 10, respectively, when using lighter loads (0.025–0.05 kg · m^2^). Notably, eccentric overload diminished earlier under heavier loads: after repetitions 8, 6, and 7 for 0.05, 0.075, and 0.100 kg · m^2^ inertial resistance, respectively [24].

Taken together, these findings suggest that the 8-repetition format used in the current study at an inertial load of 0.075 kg · m^2^ may not have induced sufficient fatigue to elicit between-group differences. Nevertheless, the CLUS protocol introduced intra-set rest and a submaximal-to-maximal repetition sequence: the initial two repetitions served to build momentum submaximally, followed by two maximal effort repetitions. Despite this additional submaximal work, the CLUS group showed no decline in power output compared to the TRAD group. These results underscore a potential advantage of the CLUS strategy, in that it may preserve training quality by mitigating fatigue while sustaining high power output. This makes cluster sets a potentially useful approach when the training goal is to maximize neuromuscular performance without compromising recovery or increasing fatigue accumulation.

### Muscle damage and biochemical response

Based on the findings related to subjective muscle soreness, both the TRAD and CLUS protocols induced soreness across multiple muscle regions, with a significant main effect of time observed at all assessed sites. Similarly, previous studies have demonstrated that FW squat training whether performed as 4 sets of 6 repetitions [4] or 10 sets of 10 repetitions [2] can induce substantial lower-limb muscle damage. Shimizu et al. reported persistent muscle soreness in the vastus lateralis and vastus medialis from Day 1 to Day 3 following eccentric FW squat exercise, particularly in non-athletic individuals [2].

Furthermore, both the TRAD and CLUS protocols induced increased stiffness in the latissimus dorsi, Achilles tendon, and patellar tendon. Notably, the TRAD group exhibited an increase in quadriceps stiffness immediately post-exercise, whereas the CLUS group maintained relatively stable values. At 24 and 48 hours post-exercise, quadriceps stiffness in the CLUS group was lower than that in the TRAD group. This pronounced increase in muscle stiffness following resistance training is associated with the degree of muscle damage [25], a finding supported by the elevated serum CK levels observed in the present study. Additionally, both protocols elicited an immediate increase in patellar tendon stiffness; however, the TRAD group exhibited greater stiffness than the CLUS group. This may be explained by the TRAD protocol’s continuous and cumulative mechanical loading without recovery intervals, which could lead to a transient decrease in tendon water content and subsequent reduction in tendon elasticity [26, 27], and the resulting increase in tendon tension may contribute to the observed rise in patellar tendon stiffness. Moreover, the lack of recovery in the TRAD protocol may have induced greater tendon microtrauma or structural fatigue, ultimately leading to a reduction in stiffness 48 hours post-exercise. In contrast, the intermittent design of the CLUS protocol likely mitigated excessive fatigue and prevented acute overloading, thereby avoiding the pronounced stiffness response observed in TRAD. Instead, the CLUS group exhibited a delayed increase in tendon stiffness at 24–48 hours post-exercise, which may reflect adaptive tendon remodeling and increased collagen synthesis [28, 29]. These findings suggest that applying FW eccentric training in a CLUS protocol may be a more effective preconditioning strategy to enhance the repeated bout effect and reduce subsequent exercise-induced injury.

Moreover, both TRAD and CLUS protocols led to significant increases in serum CK activity at 24 and 48 hours post-exercise, confirming that FW eccentric training induces muscle microtrauma a known stimulus for physiological adaptation [2]. Notably, CK levels at 24 and 48 hours were significantly lower in the CLUS group compared to the TRAD group, suggesting a more favorable muscle recovery response. This attenuated CK response may be attributed to the intra-set rest intervals in the CLUS protocol, which have been shown to reduce metabolic fatigue and neuromuscular strain [5]. These rest intervals likely enhance within-set recovery, limit cumulative muscle damage, and enable athletes to tolerate greater training volumes with reduced tissue stress.

From a practical perspective, the reduced muscle damage observed with the CLUS protocol supports its use during phases requiring high training frequency or in-season maintenance, where optimized recovery is critical. These results align with prior research indicating that cluster set structures help preserve training quality and attenuate declines in mechanical performance, potentially by minimizing neuromuscular disruption, lowering metabolic stress, and maintaining contractile function [30].

### Cardiovascular and arterial function

The present study examined the acute and delayed effects of TRAD versus CLUS resistance training on vascular and hemodynamic responses in trained individuals. The main effects of time on MAP and HR further support the distinct physiological loading patterns. Following both the TRAD and CLUS protocols, MAP decreased and HR increased at Post-0, with a gradual recovery observed thereafter. Notably, a significant time × protocol interaction was found for both DBP and CAVI, highlighting distinct cardiovascular effects between training modalities. For DBP, TRAD elicited an immediate post-exercise decrease. Similarly, CAVI, a marker of arterial stiffness, was reduced immediately after TRAD but returned to baseline thereafter, while CLUS elicited no significant changes. These transient reductions in DBP and CAVI following TRAD may reflect acute improvements in vascular compliance and a temporary reduction in arterial load. Importantly, the absence of sustained elevations suggests that TRAD does not impose adverse cardiovascular stress. While these findings may offer mechanistic insight into the acute vascular responses to different eccentric loading strategies, it is important to interpret the results within the limitations of a single-session design involving healthy young adults. Although these short-term vascular changes may appear promising, any suggestion of clinical relevance, such as potential applications to hypertension management or vascular health, should be made with caution and requires validation through longitudinal studies in clinical populations [31].

The transient reduction in CAVI post-TRAD may reflect acute arterial dilation, but its subsequent elevation suggests a reactive increase in vascular tone, potentially from prolonged muscle tension or elevated inflammatory responses [32]. In contrast, CLUS training appeared to preserve arterial function without provoking notable fluctuations, highlighting its advantage in cardiovascular stability. Notably, the immediate post-exercise reductions in blood pressure and CAVI observed in both conditions may be attributed to exercise-induced increases in shear stress, which can enhance nitric oxide (NO) production and potentially improve flow-mediated dilation (FMD) [33].

The acute cardiovascular effects of inertial FW resistance training whether localized or whole-body remain inconclusive. One study examined a FW squat protocol (2 sets of 7 repetitions) across three inertial loads (0.025, 0.05, and 0.075 kg · m^2^) and found that higher inertial loads appear to impose a greater cardiovascular burden [34]. However, in a follow-up study, high-load FW exercise (0.075 kg · m^2^) in healthy, active men did not impair brachial artery vasodilation [15]. Moreover, compared to traditional isotonic resistance training, long-term FW training has been shown to induce more favorable cardiovascular adaptations [14]

In contrast, whole-body resistance exercises seem more likely to elicit immediate post-exercise hypotension (PEH). For example, Wang et al. reported that a whole-body, high-intensity, short-rest resistance training protocol involving back squats, bench press, and deadlifts at 75% RM, led to acute reductions in blood pressure, mean arterial pressure, and ankle–brachial index [13]. Similarly, Rezk et al. [35] compared low-intensity (3 sets of 20 repetitions at 40% 1RM) and high-intensity (3 sets of 10 repetitions at 80% 1RM) whole-body resistance training across six exercises (bench press, 70° leg press, lat pulldown, leg curl, biceps curl, and 40° hip angle leg press). Both intensities resulted in post-exercise systolic blood pressure reductions, but only the low-intensity protocol significantly decreased DBP [35].

Collectively, these findings suggest that, compared to CLUS FW protocols, TRAD FW resistance training may more effectively and acutely reduce DBP and CAVI, while both protocols result in immediate reductions in MAP. These acute hemodynamic changes may provide vascular protective effects by limiting blood pressure fluctuations and arterial stiffness, with potential practical implications for resistance training programming, particularly in populations at risk for cardiovascular dysregulation.

### Central fatigue and cognitive performance

Despite an increase in perceived fatigue immediately after exercise in both groups, fatigue scores returned to baseline more rapidly in both protocols. Additionally, both groups demonstrated improvements in reaction time on the Stroop congruent and incongruent tasks at 24 and 48 hours post-exercise, suggesting potential benefits to cognitive function.

Previous research has shown that performing high-intensity resistance training (6 sets of 10 repetitions of barbell back squats at 80% 1RM with 2-minute rest intervals) may facilitate performance on simple cognitive tasks but impair more complex cognitive processes [36]. Similarly, Chang et al. reported that moderate-intensity exercise combining resistance and walking (30% 1RM and 50–60% heart rate reserve) improved Stroop test performance, whereas high-intensity full-body resistance (80% 1RM, 7 exercises) or aerobic training (30-minute running at 80% heart rate reserve) reduced prefrontal cortex tissue oxygenation and impaired cognitive function [37]. Furthermore, in older adults, eccentric exercise has been shown to produce more acute enhancements in cognitive function than concentric exercise [9]. This may be due to the greater mental demands and increased fronto-parietal network activation associated with eccentric contractions. Eccentric movements appear to require distinct neural control mechanisms, particularly within the contralateral motor cortex, compared to concentric movements [12].

Moreover, research investigating both the immediate and delayed effects of acute exercise on cognitive performance remains limited. Chou et al. reported that a single session of moderate-intensity resistance exercise (70% 1RM, involving seven exercises) significantly improved Stroop test performance and produced sustained benefits on executive control for at least 40 minutes [38]. Similarly, Barella et al. found that moderate-intensity treadmill exercise (60% HRR) led to immediate improvements in Stroop color test performance, with these cognitive gains persisting for up to 60 minutes post-exercise [39]. Furthermore, Brush et al. examined the effects of fullbody resistance exercise at varying intensities in young women and found that cognitive function improved 15 minutes after high-intensity exercise and up to 180 minutes following low- and moderateintensity exercise [40].

In the present study, full-body FW resistance exercise was found to primarily influence perceived fatigue and handgrip strength immediately after exercise. However, CMJ performance showed no significant changes at either 24 or 48 hours post-exercise. This pattern suggests that the FW protocol likely elicited a moderate-intensity exercise stimulus. Given its acute effects on physical performance and the known relationship between moderate-intensity exercise and enhanced cognitive function, it is plausible that this protocol may have contributed to cognitive improvements that persisted for up to 48 hours post-exercise.

### Limitations

The results of the present study should be interpreted with the following limitations. Firstly, the dietary intake of participants was not controlled in this study, which could have influenced the rate of recovery experienced by participants. Secondly, the load used for all participants was held constant. While groups were matched for baseline physical performance, it is likely some participants were performing their repetitions closer to failure than others, which would have impacted the amount of fatigue and muscle damage experienced between participants. Finally, while adequately powered, it should be noted that this study was performed on a homogeneous group of elite athletes. As such, it is not clear whether these results will generalize to other populations.

## CONCLUSIONS

This study examined the immediate effects of two full-body FW eccentric training protocols, traditional (TRAD) and cluster set (CLUS), on muscle damage, vascular responses, cognitive performance, and strength. While both approaches produced similar power outputs, the CLUS protocol led to smaller increases in serum CK levels and muscle/tendon stiffness, suggesting a less pronounced muscle damage response. Vascular indicators such as CAVI and DBP were better maintained following CLUS, pointing to potential benefits for cardiovascular regulation. Both groups showed improvements in cognitive performance over time, which may reflect the favorable neurocognitive impact of moderate-intensity FW training. Overall, these findings suggest that CLUS may offer a more recovery-supportive training option, particularly in settings where minimizing fatigue and tissue strain is critical.

### Highlights

1. Lower Muscle Damage: CLUS significantly reduced CK levels and showed less increase in quadriceps and tendon stiffness compared to TRAD.
2. Better Vascular Preservation: CLUS maintained vascular parameters (CAVI and DBP) more effectively, while TRAD showed only transient reductions.
3. Cognitive Benefit: Both protocols improved cognitive performance over time.
